# Age-related glomerular loss in patients with IgA nephropathy

**DOI:** 10.1093/ckj/sfaf111

**Published:** 2025-04-16

**Authors:** Hirokazu Marumoto, Nobuo Tsuboi, Takaya Sasaki, Yusuke Okabayashi, Kotaro Haruhara, Go Kanzaki, Takashi Yokoo

**Affiliations:** Division of Nephrology and Hypertension, Department of Internal Medicine, The Jikei University School of Medicine, Tokyo, Japan; Division of Nephrology and Hypertension, Department of Internal Medicine, The Jikei University School of Medicine, Tokyo, Japan; Division of Nephrology and Hypertension, Department of Internal Medicine, The Jikei University School of Medicine, Tokyo, Japan; Division of Nephrology and Hypertension, Department of Internal Medicine, The Jikei University School of Medicine, Tokyo, Japan; Division of Nephrology and Hypertension, Department of Internal Medicine, The Jikei University School of Medicine, Tokyo, Japan; Division of Nephrology and Hypertension, Department of Internal Medicine, The Jikei University School of Medicine, Tokyo, Japan; Division of Nephrology and Hypertension, Department of Internal Medicine, The Jikei University School of Medicine, Tokyo, Japan

To the Editor,

Elderly patients with immunoglobulin A nephropathy (IgAN) show clinicopathological features associated with poor kidney outcomes, possibly due to age-related declines in glomerular number and function. However, this remains untested clinically owing to methodological limitations. We recently reported estimates of the number of glomeruli per kidney and single-nephron parameters in IgAN patients [1]. As a post hoc analysis of our previous study, we conducted a cross-sectional study to elucidate the relationship between age and nephron-level parameters to determine the effect of aging on these measurements. Detailed methods for measuring the number of glomeruli per kidney and calculating single-nephron parameters have been described previously [1].

This retrospective study included 245 adult Japanese patients diagnosed with primary IgAN by a native kidney biopsy at our hospital between 2007 and 2017. The patients were the same as those in our previous study [1]. Supplemental Table S1 compares total and single-nephron parameters by age group. Figure 1 shows the relative differences in nephron-level parameters among age groups. For each parameter, relative differences from the respective mean values for the younger age group (18–29 years) were calculated to examine trends by age group. With aging, both total glomeruli (the combined number of globally and non-globally sclerotic glomeruli) and non-globally sclerotic glomeruli showed a significant decreasing trend. The total estimated glomerular filtration rate (eGFR) decreased linearly with age, whereas the single-nephron eGFR remained consistent across age groups. Both total and single-nephron proteinuria showed an increasing trend with age. The linear regression model estimated a significant annual decline with the age of 8,700 total glomeruli, including globally sclerotic glomeruli and 11,500 non-globally sclerotic glomeruli per kidney.

**Figure 1:**
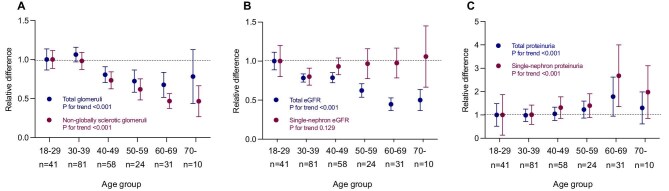
Relative differences in nephron-level parameters among age groups. Relative differences among the age groups for **(A)** total glomeruli per kidney versus non-globally sclerotic glomeruli per kidney, **(B)** total eGFR versus single-nephron eGFR and **(C)** total proteinuria versus single-nephron proteinuria are shown. For each parameter, the relative differences from the respective mean values for the younger age group (18–29 years) were calculated to examine trends by age group. Error bars indicate 95% confidence intervals. Differences among the groups were analysed using the Jonckheere–Terpstra test.

Regardless of the cause, globally sclerotic glomeruli resorb over time and eventually disappear without a trace [4]. As observed in our IgAN cohort, a decrease of 8,700 glomeruli per year in the total number of glomeruli per kidney may represent a complete loss. The annual loss of 11,500 non-globally sclerotic glomeruli per kidney in IgAN patients is clearly greater than the 6,200–6,400 loss per year reported in kidney transplant donors [1–3]. These findings suggest that the process of glomerular loss is accelerated in patients with IgAN relative to those with normal aging. In our cohort of patients with IgAN, there was a dissociation between the single-nephron eGFR and single-nephron proteinuria in their age-related changes. Interestingly, the small effect of age on single-nephron eGFR was consistent with that reported in a previous study of kidney transplant donors [5]. The increase in total and single-nephron proteinuria with age may indicate vulnerable changes in the glomeruli in elderly patients with IgAN.

The major limitations of this study include the inability to discuss causal relationships, as it was a cross-sectional study, and the reliance on needle biopsy, which may have introduced a sampling bias. The measured GFR was not available because this measurement is not routinely performed in the clinical care of patients with IgAN. Because estimates of single-nephron parameters are global averages, this method does not allow the assessment of variability in filtration function or destruction of individual glomeruli. This may be of particular concern in cases of IgAN, a disease characterized by heterogeneous glomerular lesions. Finally, it should be noted that estimates of glomerular loss cannot be individualized, as the severity of IgAN varies considerably among patients.

In summary, our findings indicate that patients with IgAN experience more glomerular loss than expected in normal aging and the remaining glomeruli become increasingly vulnerable with age. Further studies based on current findings will provide additional insights into age-related transitions in the pathophysiology of IgAN.

## Supplementary Material

sfaf111_Supplemental_File
