# Supplementary material for: Age-related glomerular loss in patients with IgA nephropathy
Source: Clin Kidney J. 2025 Apr 16;18(5):sfaf111. doi: 10.1093/ckj/sfaf111 (PMC12080221; doi:10.1093/ckj/sfaf111)
Supplement: sfaf111_Supplemental_File [file sfaf111_supplemental_file.pdf]

Supplemental Materials

Supplemental Table S1. Comparison of nephron level parameters among different age groups of patients with IgA nephropathy

Age group; years	18-29	30-39	40-49	50-59	60-69	70-	P value*
Number of patients	41	81	58	24	31	10	
Total glomeruli; per kidney	976,000 ±423,000	1,037,000 ±416,000	786,000 ±379,000	706,000 ±328,000	660,000 ±425,000	764,000 ±470,000	<0.001
Non-globally sclerotic glomeruli; per kidney	843,000 ±429,000	828,000 ±420,000	618,000 ±350,000	525,000 ±270,000	394,000 ±220,000	393,000 ±235,000	<0.001
Total eGFR; mL per min	82.5±29.6	64.6±38.9	56.7±20.4	51.4±16.8	36.9±18.5	41.4±15.7	<0.001
Single-nephron eGFR; nL per min	61.2±38.9	49.0±30.2	57.1±28.4	59.2±27.6	59.7±36.9	64.8±33.5	0.129
Total proteinuria; mg per day	584 [337-940]	660 [365-1217]	959 [481-1528]	1,296 [850-1,816]	1,287 [523-2,808]	1,124 [683-2,517]	<0.001
Single-nephron proteinuria; ng per day	372 [158-1,005]	395 [198-994]	772 [346-2,364]	1,508 [638-2,714]	2,491 [709-3,562]	1,891 [751-4,962]	<0.001

Values are presented as mean ± standard deviation or median [interquartile range]. *Differences among groups were analyzed using the Jonckheere Terpstra test. eGFR, estimated glomerular filtration rate; IgA, Immunoglobulin A
